# Editorial: Insights in sex and gender in cardiovascular medicine: 2023

**DOI:** 10.3389/fcvm.2024.1489728

**Published:** 2024-09-30

**Authors:** Daniela Trabattoni

**Affiliations:** Unit of Interventional Cardiology, Monzino Cardiology Center (IRCCS), Milan, Italy

**Keywords:** gender differences, cardiac disease, acute coronary syndromes, heart and mind, clinical outcomes, interventional procedure

**Editorial on the Research Topic**
Insights in sex and gender in cardiovascular medicine: 2023

In 2022, the 10th IGM (International Society of Gender Medicine) Congress clearly highlighted how attention to sex and gender is relevant in the biological, psychosocial and clinical aspects of all medical specialties. Furthermore, the relevance of developing strategies against discrimination and inequalities emerged and, the importance of politics in taking into account sex and gender differences, was also emphasized.

So, based on the various competences, going from endocrinology to cardiology and pneumology, from medical sociology, psychology to geriatric medicine and surgery, the main objective is to face the transversal dimension of the gender-specific medicine and to improve the understanding of sex and gender differences. To this end, has been built-up this focus topic with the key objective of encouraging gender-specific clinical and basic research and transferring the results into clinical healthcare.

The topic issue has collected 6 research studies analyzing women specific outcomes after cardiac diseases and cardiac surgery, the link between heart and mind in CV diseases, and the role of AI predicting clinical outcomes in women after acute coronary syndromes.

Previous studies have established that gender disparities in mortality are influenced by social, behavioral, and epidemiological factors. In the study conducted by Lv et al. on data from the National Health and Nutrition Examination Survey (NHANES) collected between 2005 and 2018, a total of 38,924 participants were included. Consistent with previous findings, significant gender differences in mortality rates were found within the U.S. population. Addressing and reducing the gender mortality gap is of utmost importance in promoting equitable access to evidence-based healthcare and preventive measures. Worse clinical outcomes after cardiac surgery and need for in-hospital readmission have been described especially in women. The single centre retrospective observational DREAMS (Database for REAdmission after Major cardiac Surgery) registry including all patients after major cardiac surgery from January 2012 to September 2020 in Switzerland, by Koechlin et al. focused on sex-specific differences regarding underlying factors for readmissions after major cardiac surgery. This study did not find a statistically significant difference in the overall cohort and neither in the subgroup analysis including patients after CABG surgery only, or isolated aortic valve replacement. Infection were the most common reasons for readmission in both sexes. The analysis of differences in reasons for readmission are important for individualized medicine approaches. Mortality and complications rates among females with ACS are significantly higher than in males and the role of machine learning algorithms for prediction of clinical outcomes among females has been investigated to help mitigate sex-biases. Additionally, the study by Shankhwar et al. explored the influence of menstrual phases on cardiovascular and autonomic responses in both resting and during the central hypovolemia induced by lower body negative pressure (LBNP). The results obtained demonstrated how central hypovolemia leads to increased cardiovascular and autonomic responses, particularly during the luteal phase of the menstrual cycle, likely due to higher estrogen levels and increased sympathetic activity. In a cardiovascular preventive approach, the research by Giuliani et al. focuses on evaluating the potential impact of psychological well-being on the risk of developing cardiovascular disease in women who have no prior history of such conditions, over a 10-year period. Surprisingly, the findings indicate that psychological well-being is a stronger predictor than traditional cardiac risk factors (such as hypertension, diabetes, and smoking) for the development of cardiovascular disease within a decade, among women with no prior history of cardiac disease.

Finally, the retrospective analysis by Bonanni et al. has definitely filled the knowledge gap on gender-differences in acute and long-term outcomes after left atrial appendage closure, showing comparable rates of all-cause mortality, stroke, transient ischemic attack and major bleeding in men and women 12 months after the procedure.

